# Optimized surgical treatment and study management for oncology patients in East Saxony (MISSION4Sax): study protocol of a multicentre pilot study

**DOI:** 10.1007/s00432-026-06599-2

**Published:** 2026-09-02

**Authors:** Christin Richter, Anne Döring, Floris Berg, Michelle Roost, Andreas Hasselberg, Carola Martin, Hannes Schlieter, Daniela Piontek, Olaf Schoffer, Jochen Schmitt, Jürgen Weitz, Grit Krause-Jüttler, Johanna Kirchberg

**Affiliations:** 1 Department for Visceral, Thoracic and Vascular Surgery, Faculty of Medicine and University Hospital Carl Gustav Carus, TUD Dresden University of Technology, Dresden, Germany; 2 Center for Evidence-Based Healthcare, Faculty of Medicine and University Hospital Carl Gustav Carus, TUD Dresden University of Technology, Dresden, Germany; 3 Research Group Digital Health, Faculty of Business and Economics, TUD Dresden University of Technology, Dresden, Germany; 4 National Center for Tumor Diseases (NCT), NCT/UCC Dresden, a partnership between, DKFZ, Faculty of Medicine and University Hospital Carl Gustav Carus, TUD Dresden University of Technology, and Helmholtz-Zentrum Dresden-Rossendorf (HZDR), Dresden, Germany

**Keywords:** Surgical oncology, Multidisciplinary tumour board, Patient pathways, Health services research, Networks, Health services accessibility

## Abstract

**Purpose:**

MISSION4Sax aims to develop and pilot new concepts for cross-sectoral oncological care, data integration and networking between maximum care centres and rural practitioners in medical underserved, enabling access to guideline-based therapy, innovative surgery, and clinical trials.

**Methods:**

This pilot project is an exploratory multicentre, prospective longitudinal study. It assesses the feasibility, acceptability, implementation and data-quality aspects of the proposed care model and study infrastructure, rather than to determine the effectiveness or causal impact of the interventions. It implements an interdisciplinary, cross-sectoral care model centred on a regional surgical indication tumour board at the National Center for Tumor Diseases (NCT) Dresden. Evidence-based, standardised patient care pathways are developed and piloted across three regional hospitals and two oncology practices. A database using the infrastructure of the NCT Core Unit “Registry Trial Platform”, targeted training programmes and “Flying Data Nurses” support coordination, data flow and communication. Patient-reported outcomes complement the clinical data. Routinely collected health insurance data will be used exclusively as external reference data for a descriptive comparison with the MISSION4Sax cohort; no individual-level data linkage will be performed. A mixed-methods process evaluation will be performed alongside the project.

**Expected outcomes:**

The study will assess feasibility in routine care and explore access to specialised care, waiting times, coordination, pathway adherence, and clinical and patient-reported outcomes.

**Conclusion:**

Strengthening cancer care infrastructure, interdisciplinary cooperation, and digital systems may support equitable access to high-value care in rural regions. The proposed tumour board, patient pathways, and trained staff may improve care quality and collaboration across sectors.

**Trial registration:**

DRKS00036134 (registration date: 30 June 2025)

## Introduction

The World Health Organization forecasts a 77% increase in new cancer cases worldwide by 2050 (WHO 2024). A significant increase to around 600,000 cases per year is also expected in Germany, accompanied by a shift in tumour entities (Baumann et al. 2022; Stock et al. 2018). The optimal care of oncological patients requires a multimodal, interdisciplinary approach, as established at Comprehensive Cancer Centers (CCC) and within the National Center for Tumor Diseases (NCT) network. For patients with solid tumours, timely access to specialised surgical expertise is an essential component of multimodal cancer care. However, access to specialised surgical oncology services remains uneven, particularly in rural regions such as Eastern Saxony, where the concentration of surgical expertise is limited (Datta et al. 2024; Gutacker et al. 2024; Nimptsch and Mansky 2017; Spreider et al. 2024). To provide coordinated surgical oncological care that is tailored to patients’ needs while making efficient use of available resources, close collaboration between the CCCs and regional stakeholders is required (Brandts 2019).

The need for coordinated oncological care between CCCs and regional healthcare providers is particularly pronounced in Eastern Saxony, where low population density and limited healthcare accessibility pose additional challenges for patients requiring specialised cancer treatment. The project region comprises the districts of Bautzen, Görlitz, and Sächsische Schweiz-Osterzgebirge, with population densities of 116–148 inhabitants/km^2^, compared with 1722 inhabitants/km^2^ in Dresden, where the CCC is located (Statistisches Landesamt des Freistaates Sachsen 2025). Reflecting these structural differences, 94.36% of Dresden’s population live within 1 km of a general practitioner practice, compared with only 61–68% in the rural districts (Bundesinstitut für Bau-, Stadt- und Raumfoschung-BBSR 2025). Despite these disparities, the annual number of new cancer cases remains substantial, ranging from 2084 to 2468 in the rural districts and reaching 3939 in Dresden in 2023 (clinical counting method) (Krebsregister Sachsen gGmbH 2025).

### Identification of key challenges

In response to persistent disparities in access to high-quality oncological care and the fragmentation of care pathways across East Saxony, the pilot project “Optimized surgical treatment and trial management of oncological patients in East Saxony” (MISSION4Sax) addresses four key challenges:

1. Lack of cross-sectoral care pathways: Although S3 guidelines on oncological care exist, there is a lack of nationwide, coordinated implementation outside of certified centres. Outpatient and inpatient care providers are often inadequately connected (Landesverbände der Sächsischen Krankenkassen et al. 2024; Langer and Follmann 2015; Müller et al. 2023; Schaefer et al. 2015).
2. Limited access to surgical expertise of a CCC: In rural regions such as Eastern Saxony, access to specialised surgical oncology services is limited, which is associated with worse clinical outcomes (Datta, et al. 2024; Gaber and Wildner 2011; Gutacker et al. 2024; Schoffer et al. 2022).
3. Insufficient data integration: A centralised, detailed, and comprehensive registry for clinical, laboratory-based, biomarker-related and patient-reported data is still lacking. The informative value of existing cancer registries is limited from a scientific perspective (Arndt et al. 2020; Loos et al. 2025).
4. Limited access to clinical trials and knowledge transfer: Patients in rural regions participate significantly less frequently in clinical trials, despite their potential to significantly improve prognosis (Berger-Thuermel et al. 2023; Schoffer et al. 2022). Communication and knowledge transfer between maximum care centres and rural practitioners are often deficient (Gutacker et al. 2024; Landesverbände der Sächsischen Krankenkassen et al. 2024; Loos et al. 2025; van den Berg et al. 2021).

### Study objectives

The overall goal of MISSION4Sax is to ensure that patients have access to guideline-based therapy, clinically indicated innovative surgical procedures, and clinical trials, regardless of the place of treatment or residence.

To achieve this goal, MISSION4Sax develops and pilots an integrated, cross-sectoral care model that incorporates new concepts for cross-sectoral care, data integration and networking between maximum care centres and rural practitioners, while considering the current German hospital reform (KHVVG 2024; KHAG 2026).

The project comprises four complementary, interrelated, and synergistic sub-projects (SP). Together, these sub-projects address the project’s goals and the mentioned key challenges. Each SP focuses on specific objectives and a central research question, as outlined below:

#### SP 1: Guideline-based and intersectoral patient pathways

*Objective*: Development and evaluation of standardised care pathways to optimise the coordination and quality of surgical oncological treatment.

*Research question*: How does the development and implementation of guideline-based patient pathways influence the coordination of oncological care in Eastern Saxony?

#### SP 2: Cooperative surgical indication tumour board (CoSITuB)

*Objective*: Establishment of a virtual tumour board to promote access to modern surgical procedures and ensure the quality of treatment decisions.

*Research question:* Does the CoSITuB enable improved access to modern surgical procedures and clinical trials for cancer patients in rural areas?

#### SP 3: Database and process evaluation

*Objective*: Setting up a database as groundwork for a regional registry to systematically analyse patient data, care processes and implementation parameters.

*Research question:* How can the study cohort be characterised? How are the interventions implemented and accepted?

#### SP 4: Flying data nurses (FDNs) and training programme

*Objective*: Introduction of mobile data managers and a training programme to improve networking, knowledge of modern techniques, clinical trial operations, and study integration.

*Research question:* What is the impact of FDNs, training, and exchange formats on improving study knowledge, increasing access to studies, and fostering interprofessional networking?

**Fig. 1:**
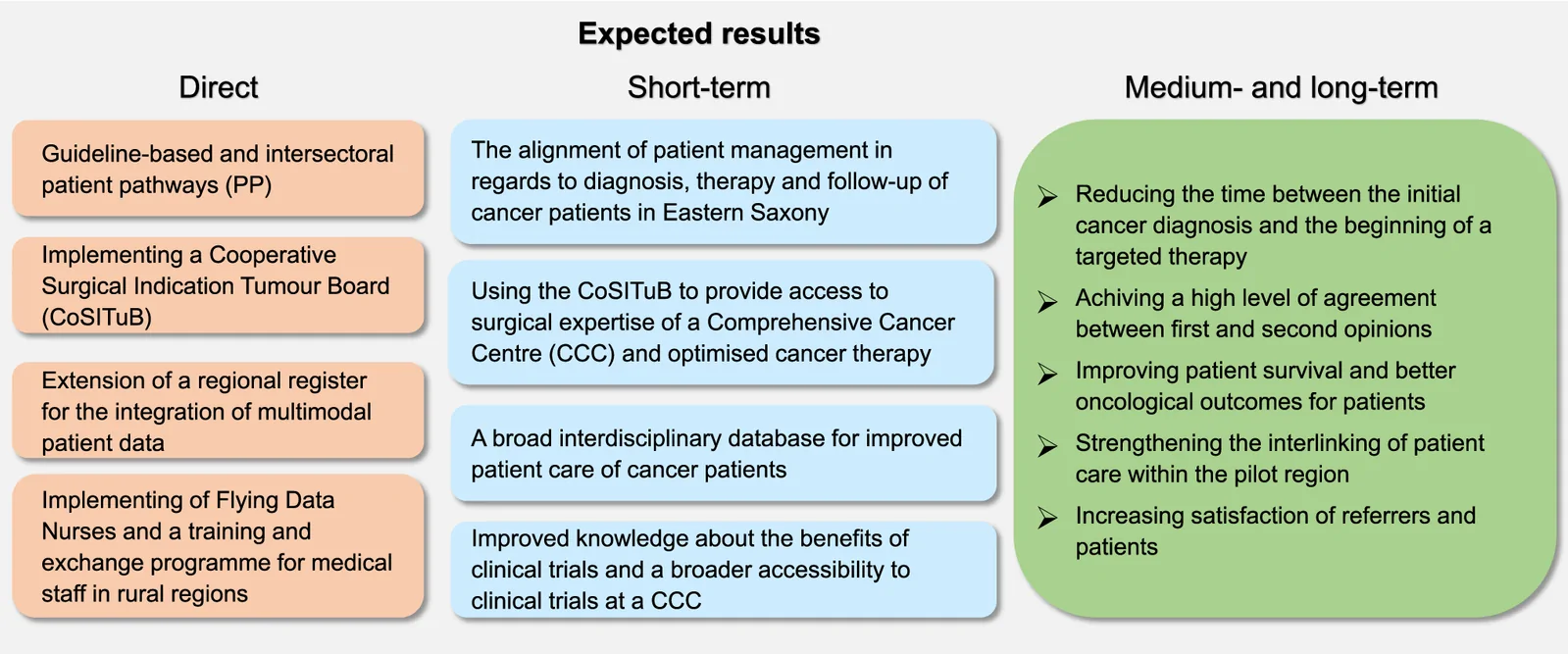
Expected direct, short-, medium- and long-term results of MISSION4Sax

This project will develop evidence-based approaches to sustainably optimise surgical oncological care in the Eastern Saxony region by systematically evaluating the acceptance and feasibility of new care models. In doing so the project contributes to the core objectives of the National Decade Against Cancer (2019–2029): equitable access to care and close links between research and clinical practice. The aim is to ensure that all cancer patients in Germany have equal access to high-quality oncological care and innovative treatments, regardless of where they live (Loos et al. 2025). Figure 1 illustrates the expected direct, short-, medium- and long-term results.

## Methods

### Study design and procedure

MISSION4Sax is designed as a non-controlled, exploratory, multicentre, prospective longitudinal feasibility study. The project will be carried out in collaboration with five pilot partners over a period of 36 months. The partners consist of three hospitals (standard and specialist care, with 145, 237 and 390 inpatient beds) and two oncology practices (with around 1350–2000 patients per quarter) serving as local study sites in Eastern Saxony. Following approval by the Ethics Committee, a twelve-month pilot phase will begin, followed by a twelve-month stabilisation phase. Recruitment starts at the beginning of the pilot phase, the last patient will be enrolled nine months before the end of the project, and the final data collection will occur six months before the end of the project.

**Fig. 2:**
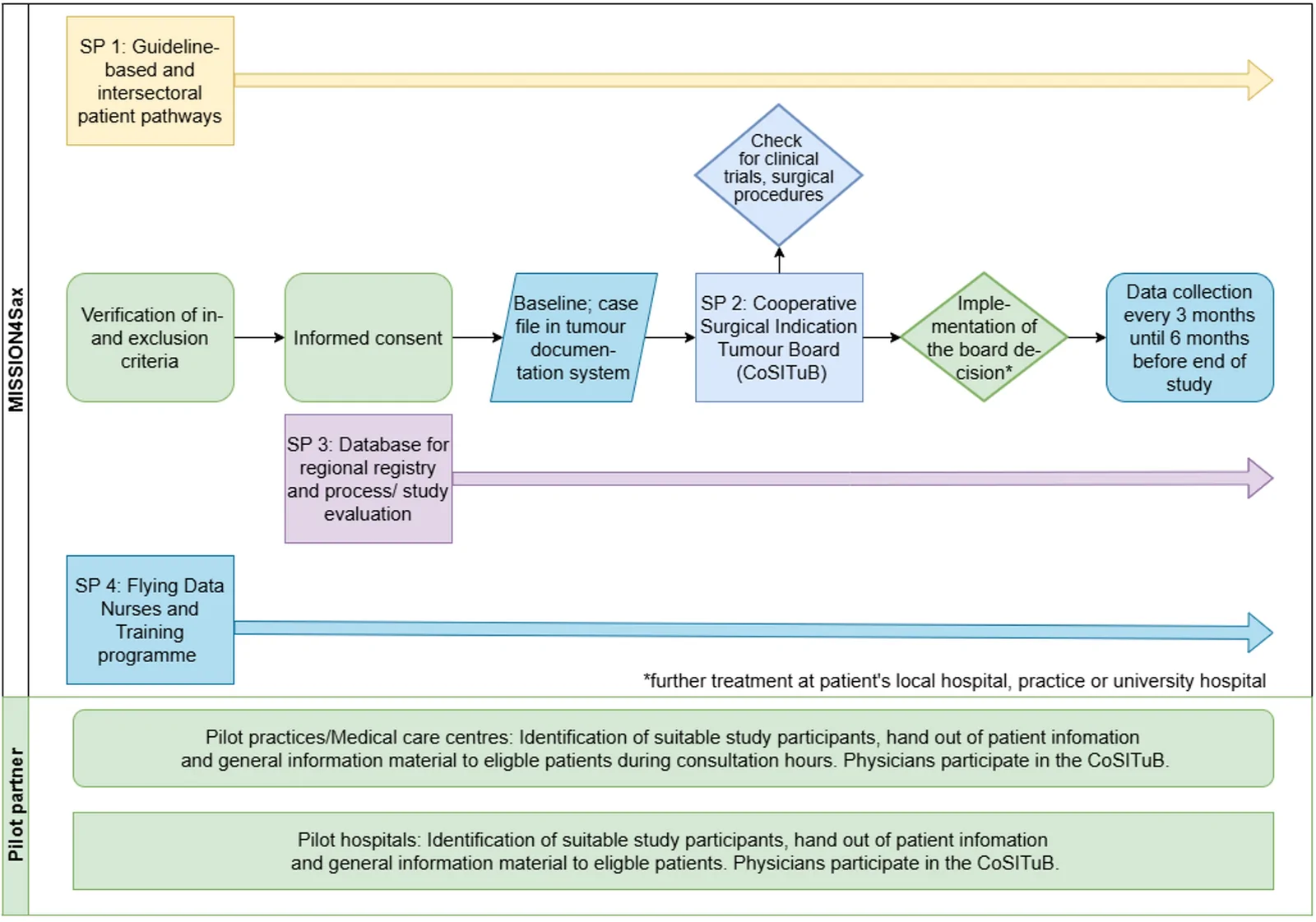
Planned course of study during the pilot phase

The four sub-projects (SP 1–4) are closely interlinked in terms of content (see Fig. 2). FDNs (SP 4) accompany the enrolled patients along defined patient pathways (SP 1). The patients’ medical cases are presented in the CoSITuB (SP 2) to discuss surgical options and clinical trials. All patients are included in a database (SP 3). In parallel, a training programme for medical staff and patients (SP 4) will be implemented.

#### SP 1: patient pathways

Structured patient pathways organise the treatment process by clearly defining interfaces, responsibilities, and transition criteria. They promote transparency and quality through documentation-based process monitoring and facilitate shared decision-making. A simplified version will be provided for patients to enhance their understanding of the treatment process within the project and encourage active participation.

#### SP 2: cooperative surgical indication tumour board (CoSITuB)

The aim is to establish a virtual, weekly interdisciplinary tumour board at the NCT/UCC Dresden to evaluate complex surgical cases. Data is provided by the FDNs via the Tumour Documentation System (TDS). In addition to cross-sectoral therapy decisions, this enables early access to innovative studies.

The CoSITuB is a multidisciplinary tumour board comprising specialists in medical oncology, surgical oncology, internal medicine, palliative care, and radiology. It complements the existing multidisciplinary tumour boards at the NCT/UCC Dresden by providing expert consultation for complex oncological cases from rural areas, with a particular focus on operability within the overall multimodal treatment strategy.

Recommendations are developed by consensus with the referring physicians. Clinical responsibility, patient communication, and follow-up procedures are agreed and documented during the meeting. Urgent cases can be referred at any time via secure communication channels and are prioritised accordingly. The final recommendation is communicated to the referring centre on the next working day, while its implementation and relevant time intervals are prospectively documented by the Flying Data Nurse. Where appropriate or in cases of disagreement, cases may be referred to a specialised tumour board for further discussion.

#### SP 3: database & IT structures

The creation of a database based on the NCT/UCC “Registry Trial Platform” will allow for the collection of clinical data, patient-reported outcomes (PROs), histological findings, and further project-related data. This database will support descriptive analyses of the study cohort and its treatment outcomes, including complications. Furthermore, the time stamping of diagnoses and therapies permits longitudinal analyses. The IT implementation and coordination with external IT partners from collaborating hospitals and oncology practices will be managed by SP 3.

#### SP 4: training & flying data nurses (FDNs)

A training programme promotes the transfer of knowledge about studies and surgical procedures. Formats such as short lectures following the case discussions during the CoSITuB or work shadowing are offered in line with requirements. The core of SP 4 is the piloting of the FDN concept: FDNs coordinate patient recruitment, document relevant patient data in the TDS, accompany patients along the pathway, and serve as an interface to the CoSITuB. Each pilot facility acts as local trial site and is assigned a primary FDN and a deputy. The deployment is location-based and orientated towards local needs.

### Participants and recruitment

The study population consists of patients with a complex visceral oncological underlying disease, presenting at one of the five external, local trial sites in East Saxony with the following inclusion criteria:

- Complex underlying oncological disease, i.e. the extent or type of diagnosis or therapy of the oncological disease exceeds the possibilities of the respective site and requires extended external, interdisciplinary consultation on the medical case or requires the involvement of an expert centre (NCT/UCC),
- A visceral tumour entity (primary, metastases, or recurrence): Colorectal (C18, C19, C20), pancreatic (C25), oesophageal (C15), gastric (C16), biliary tract and hepatic tumours (C22.0/C22.1, C23, C24), or other rare tumour entities,
- A minimum age of 18 years,
- Ability to understand the study information and provide written informed consent,
- Ability to read and understand the German language,
- Presentation at the external trial centres.

Excluded are patients who are unable to give consent or cannot sufficiently read and understand German, as well as pregnant women. A sample size of 50–100 participants is expected. As this is a pilot feasibility study, no formal sample size calculation was performed. The anticipated sample size was determined pragmatically based on the expected recruitment estimates of the participating pilot partners and on historical case numbers during project planning.

Furthermore, the physicians at the external trial sites and the FDNs will take part in surveys as part of the process evaluation of the project.

### Primary and secondary endpoints

The primary endpoint of this project is improved access to clinically indicated, guideline-concordant surgical techniques and innovative clinical trials for cancer patients in Eastern Saxony.

As a feasibility study, the project aims to assess whether the proposed care structures and processes can be implemented successfully in routine practice. It is not designed to provide confirmatory evidence of intervention effectiveness.

Secondary endpoints are described according to the individual sub-projects in Table 1.

**Table 1:** Overview of secondary endpoints

Sub-project	Items	Variables
SP 1: Patient pathways	Clinical outcomes	Complication rate (30 and 90 days) with Clavien-Dindo-Classification Grade III+ Mortality (in hospital, 30 and 90 days) Readmission rate (30 days)
	Patient satisfaction	Perceived quality of care Treatment satisfaction at baseline and project completion Involvement in decision-making
	Interdisciplinary collaboration	Perceived quality and coordination of care processes
	Process efficiency	Average waiting and transfer times Completeness and deviations in pathway documentation Decision-making confidence
	Sample survey of the total population of oncology patients treated at one local study site	Number of patients not included in CoSITuB Diagnosis Surgical indication Mortality
	Economic efficiency	Average length of hospital stays Duplication of diagnostic procedures
SP 2: Cooperative surgical indication tumour board (CoSITuB)	Use of CoSITuB	Number and characteristics of presented cases
	Concordance	Agreement between initial and second opinion on operability and surgical approach Number of treatment modifications
	Implementation	Surgical indications and procedures (robotic / open / minimally invasive) per site Adherence to tumour board recommendations
	Timeline tracking	Time from board decision to treatment initiation and discharge summary dispatch
SP 3: Database & IT structures	Patient data	Age Sex Diagnosis (ICD-10, staging, histology) Comorbidities
	Treatment	Surgical procedures, including R0 resection (OPS-codes) Systemic therapy
	Quality of life	General health, physical, role, emotional, cognitive and social functioning (quarterly)
	Referrer satisfaction	Care pathways, CoSITuB, FDN, and the exchange/training programme
SP 4: Training & flying data nurses (FDNs)	Communication	Number and type of site visits and exchanges
	Clinical trials	Patient screening and enrolment in innovative studies
	Training programme	Utilisation and evaluation of exchange and educational activities

### Data management, data governance and data protection

The regional pilot partners will be integrated into the existing IT infrastructure at the NCT/UCC (TDS) to streamline data submission to the Saxony Cancer Registry (KRS).

Clinically relevant patient data will be extracted by FDNs from local hospital or practice systems and entered into the NCT/UCC’s TDS. Clinical recommendations are documented within the TDS and shared with partners via automatically generated physician reports. Study data not captured in the TDS will be managed via a REDCap database and study participant management (STeVe).

The TDS records data on all cancer patients presented at the NCT/UCC, allowing close monitoring of treatment and outcomes, while enabling automatic data transfer to the mandatory regional cancer registry (KRS). During the project, pilot partners are exempt from separate reporting to the KRS due to TDS integration.

The database—based on the infrastructure of the NCT/UCC Dresden Core Unit “Registry Trial Platform”—collects multimodal data including PROMs, clinical information, laboratory results, and histology. It supports decision-making, care pathway adherence monitoring, and quality assurance. All patients involved in care pathways, CoSITuB, or other interventions are included. Follow-ups are conducted quarterly via data extraction from local systems.

Before integration into the research database, clinical and questionnaire data are pseudonymised. Direct identifiers listed in Table 2, such as name and date of birth, are used only for identification, follow-up, and linkage purposes (TDS—REDCap) and are stored separately from the analytical dataset. The re-identification key is maintained separately on access-protected systems and is accessible only to authorised personnel responsible for participant management. Researchers conducting analyses receive pseudonymised data without direct identifiers. Data consistency and completeness are monitored through standardised data entry and ongoing plausibility checks.

**Table 2:** Overview of study data

The following study data will be collected via Tumour Documentation System (TDS), study participant management (STeVe) and electronic data capture software (REDCap) at baseline T_0_, after 3, 6, 9, 12 months at T_1_-T_4_, respectively 6 months before the end of the project (End of Study):
Demographic data
Last name, first name, date of birth, age, gender, family history
Health insurance data
Private or statutory health insurance
Diagnosis: carcinoma in general
Date of initial diagnosis, reason for diagnosis, diagnosis made on, diagnostic certainty, ICD-10, UICC stage, cTNM stage, lymph node involvement yes/no
Comorbidities
Start/end of diagnosis, ICD-10
Chemotherapy
Start/end of chemotherapy, type of chemotherapy (neoadjuvant/adjuvant/palliative), cycles, medication (active ingredient), dose levels in %, type of toxicity, toxicity level, date of onset of side effects, total residual tumour
Other diagnostics/ findings
Date of diagnosis, type of diagnosis, location/department, findings, ECOG performance status, date of issue of referral form (between partners), reason for referral from partner
Postoperative findings/ histology
Date of findings, pTNM stage, UICC (classification for staging), residual tumour tissue (residual tumour), pathology with findings no., number of lymph nodes (removed/affected), tumour grading (G), recurrent disease, type of metastases, date of examination for secondary metastasis, type of secondary metastasis
Therapies
Date of therapy, type of therapy (CTx, RCTx, RTx, surgery), OPS-code
Surgeries (procedure/ course)
Date of surgery, surgical procedure (laparoscopic, robotic, open), postoperative complications according to CDC (date + type), type of treatment for the complication, highest CDC grade, reoperation in case of complications (date + type)
Tumour board case presentation/ reports (internal and external)
Date of TB presentation, reason for TB presentation, presenting physician, second opinion yes/no, category (surgical, study option, case discussion), initial opinion on resectability, second opinion on resectability, agreement between initial opinion and second opinion (yes/no), CoSITuB recommendation (surgery/CTx/RTx/no therapy/other), degree of implementation of the recommendation (fully implemented/partially implemented/not implemented), duration of: decision CoSITuB and start of the intervention, duration of: decision CoSITuB and doctor's letter, duration of: start of intervention and doctor’s letter
Hospital stays
Number, location, department, reason (causal connection to carcinoma), duration (hospital stays/ intensive care stays), readmission to intensive care unit/ reason, transfer to another hospital/ reason
Death
Date of death
Participation clinical trials
Study title, date of screenings, date of inclusion
Quality of life (EORTC-QLQ-C30)
CDC: Clavien-Dindo Classification CoSITuB: Cooperative Surgical Indication Tumour Board CTx: Chemotherapy ECOG Performance Status: Eastern Cooperative Oncology Group Performance Status for the assessment of oncology patients ICD-10: International Statistical Classification of Diseases and Related Health Problems OPS-Code: Surgery and procedure codes RCTx: Radiochemotherapy RTx: Radiotherapy TB: Tumour board TNM: Tumour, Node, Metastasis—tumour classification system UICC: Union for International Cancer Control

### Study data and data sources

At the beginning of the project, structural characteristics of the pilot facilities (e. g. sponsorship, number of beds, specialities, research activities, IT infrastructure) are documented.

Patient-related data is primarily collected from the internal hospital information systems and patient files of the pilot facilities. Table 2 shows all relevant variables. Up to six measurement points are planned for all included patients (baseline and every three months). Socio-demographic and clinical data including diagnostics, course of treatment and quality of life (EORTC QLQ-C30, validated German Version) will be recorded. Participation in CoSITuB is independent of the survey dates.

### Process data and evaluation

As part of an accompanying process evaluation (Moore et al. 2015; Skivington et al. 2021), the feasibility and impact of the interventions in the sub-projects are analysed. The assessment of intervention impact is exploratory and is intended to generate preliminary estimates and hypotheses for a subsequent effectiveness study. It is not designed to provide confirmatory evidence of effectiveness. This is based on a theory-based model of change (Theory of Change) (De Silva et al. 2014), which depicts the context, implementation, and impact mechanisms. A mixed-methods strategy combines qualitative and quantitative data (e.g., from interviews, checklists, and database data).

The implementation of the patient pathways is evaluated using structured checklists that assess deviations and completeness along defined process criteria. Additionally, to assess the impact of network-based collaboration on the standardisation of care processes, a retrospective analysis of patients from a defined timeframe at a pilot partner will be conducted at the end of the pilot phase. Data collected at project baseline and end will include demographics, diagnoses, treatment decisions, and mortality for all patients with tumour entities considered in the project. This analysis will exploratory assess whether the standardised criteria in the pathways may have contributed to more consistent referral practices and will provide an estimate of the proportion of patients treated via the CoSITuB compared to the overall cohort.

Finally, the data flows between the information systems involved are analysed to identify requirements for data completeness and quality and to develop a corresponding evaluation concept (Table 3).

**Table 3:** Data collection as part of the process evaluation

Target group	Method	T_0_	T_1_	T_2_	T_3_	T_4_	Data
Physicians	Focus group (*n*=1)			x			Patient recruitment, Acceptance, feasibility and adaptions of the CoSITuB, Impact on patient oncological care, Events for medical staff, Satisfaction of referring physicians, Decision-making confidence
	Process documentation (Contact log, Training programme, Feedback questionnaires)	x	x	x	x	x	Offer, acceptance (evaluation) and implementation of the CoSITuB, Events for medical staff
	Questionnaire: CHAT (Centre for Social Impact and Collaboration for Impact 2017)	x				x	Interdisciplinary collaboration, Perceived quality, coordination and communication of the care processes
Flying data nurses (FDN)	Focus group (*n*=1)				x		Recruitment and data collection process, Conducting the CoSITuB, Patient support, Facilitators and barriers
	Process documentation (e. g. contact log)	x	x	x	x	x	Intersectoral communication, Type and frequency of communications, Site visits
Patients	Semi-structured interviews (*n*=5)				x		Treatment satisfaction, Perceived quality of care, Informed decision-making, Decision-making confidence
	Questionnaire: FACIT-TS-PS (Peipert et al. 2014)	x				x	Satisfaction with the care process at the beginning and the end of the study
	EORTC-QLQ-C30	x	x	x	x	x	Quality of life questionnaire
	Process documentation (Training programme, Feedback questionnaires)	x	x	x	x	x	Offer, acceptance and implementation of events for patients
	Checklist	x		x		x	Patient pathway deviations and completeness

T_0_ = baseline, T_1_ = 3 months, T_2_ = 6 months, T_3_ = 9 months, T_4_ = 12 months

CHAT: Collaboration health assessment tool

CoSITuB: Cooperative surgical indication tumour board

EORTC-QLQ-C30: European organisation for research and treatment of cancer quality of life questionnaire—Core 30

FACIT-TS-PS: Functional assessment of chronic illness therapy—treatment satisfaction—patient satisfaction

### Questionnaires

Patient treatment satisfaction and quality of life will be measured using the following questionnaires, validated German versions:

- European Organisation for Research and Treatment of Cancer Core Quality of Life Questionnaire (EORTC QLQ-C30) (Aaronson et al. 1993; Fayers and Bottomley 2002)
- Functional Assessment of Chronic Illness Therapy—Treatment Satisfaction—Patient Satisfaction (FACIT-TS-PS) (Peipert, et al. 2014).

The satisfaction of participating pilot partners will be assessed using the.

- Collaboration Health Assessment Tool (CHAT) (Centre for Social Impact and Collaboration for Impact 2017).

### Focus groups and semi-structured interviews

Insights from interviews and focus groups will contribute to process evaluation and refinement of project components. Qualitative and quantitative data will be integrated through triangulation during data analysis and interpretation. Findings from interviews and focus groups will be compared with questionnaire data, process documentation, and clinical project data to provide a comprehensive understanding of implementation processes and outcomes. Therefore, two focus groups will be held, either on-site or virtually, one with physicians and tumour board members, and another with FDNs. Each focus group is expected to include approximately 5 purposively selected participants representing the different professional groups and pilot sites and will be conducted by two researchers—one moderating, the other observing and documenting key insights. Audio recordings (with informed consent) will be pseudonymised, transcribed, and later anonymised.

A semi-structured guide will frame discussions, focusing on the management of complex oncological cases and interdisciplinary collaboration. The semi-structured interview and focus group guides are based on the project objectives and needs assessment. They comprise predominantly open-ended questions with flexible prompts, allowing participants to elaborate on their views while ensuring consistency across data collection. Key themes include expectations, patient screening and recruitment, interdisciplinary collaboration within the cross-sectoral tumour board, technical implementation, clinical pathways, support by the Flying Data Nurses, communication and training, perceived benefits and barriers, and opportunities for improvement and long-term sustainability. The guides will be piloted within the project consortium, incorporating feedback from medical staff, and the patient interview guide will be pre-tested. Qualitative data collection will follow an iterative approach, with findings from the initial focus groups informing subsequent interview guides to further explore and validate emerging themes across participant groups.

Transcripts will follow a simplified system (Dresing and Pehl 2024), enabling efficient analysis while maintaining essential content features such as emphasis and pauses.

Transcripts will be analysed using qualitative content analysis (Flick 2018; Mayring 1983). The analysis combines deductive coding (from the interview guide) and inductive development of new codes. Meaning units will be extracted, categorised, and illustrated with anchor examples. The transcripts will be double-coded to ensure quality.

Additionally, guided one-on-one interviews with patients will follow the same protocols for recording, transcription, and analysis. Five purposively selected patients (one from each pilot site, representing different tumour entities) will be interviewed to capture a range of patient perspectives. Considering the exploratory purpose of the interviews, the aim of obtaining perspectives from all pilot sites, and the available resources during the pilot phase, a purposive sample of five patients was selected. This sample is intended to provide an initial range of patient perspectives and is not expected to be representative or to achieve thematic saturation. Transcripts will be coded independently by two researchers, with disagreements resolved through discussion and, if necessary, consultation with a third researcher.

## Data analysis

### Descriptive analyses

Disease- and treatment-specific variables will be described according to age group, gender, and year. This includes therapy use, treatment-related complications, and quality of life and how it changes over the course of the study (quarterly assessed). The questionnaires are evaluated according to the respective manuals (https://www.facit.org/measures/facit-ts-ps; Fayers et al. 1995). Additional indicators of service provision and utilisation will be assessed to generate a comprehensive overview of the care structure and treatment pathways. Sequences of diagnostic and therapeutic steps and the collaboration between providers will be illustrated. To this end, frequencies, proportion values with confidence intervals, and time intervals between events will be used as descriptive tools. Where appropriate, network-based methods such as Sankey charts may be used.

Missing values will be reported in terms of their extent and distribution. No imputation or interpolation will be performed, analyses will be based on the available data. The occurrence and pattern of missing values will also be used to identify potential areas for improvement in data collection and documentation in future projects.

### Further (exploratory) analyses

For exploratory analyses, categorical variables and count variables will be evaluated using the descriptive methods specified above. For metric variables, particularly time intervals, the mean, standard deviation, and range will be reported.

Based on TDS and REDCap data, relevant clinical parameters will be described, including.

- Surgical approach (open/minimally invasive/robotic)
- Hospital and Intensive Care Unit stay duration after oncological surgery
- Revision surgeries (30 days) and postoperative complications with Clavien-Dindo-Classification Grade III+ (30 and 90 days)
- Hospital readmissions (30 days)
- Time from initial diagnosis to treatment
- Number of R0 resections and lymph nodes removed
- Referral to a university hospital or certified cancer centre (if applicable)

Patient satisfaction will be analysed using validated questionnaires. Partner satisfaction will be assessed through qualitative analysis of focus groups.

The potential to use registry platform data for further in-depth analyses will be assessed over the course of the study.

### Routine health insurance

Routine health insurance data from AOK PLUS, which has broad coverage in Saxony, will be used as external reference data. These data will not be obtained for individual MISSION4Sax participants and will not be linked to the MISSION4Sax database. Instead, aggregated disease characteristics and, where available, selected care-related indicators will be compared descriptively with corresponding data from the AOK PLUS-insured population.

## Discussion

The aim of this project is to develop and pilot new concepts for cross-sectoral oncological care, data integration, and networking between maximum-care centres and rural practitioners in medically underserved areas with limited resources. The project seeks to improve patient access to guideline-based therapies, innovative surgical procedures, and clinical trials, with primary focus on the feasibility and acceptability of the combined approach. This cross-sectoral and interdisciplinary approach enhances the alignment and coordination of regional cancer care, particularly by facilitating multidisciplinary decision-making, harmonizing surgical pathways, and ensuring that complex oncological procedures are planned and delivered within coordinated networks of specialised providers (CraNE Joint Action 2024). Establishing a regional surgical indication tumour board (CoSITuB), standardised patient pathways, and a database as a foundation for a regional register supported by Flying Data Nurses (FDNs) represents a novel approach to optimising care coordination.

The study is not expected to interfere with standard care processes for patients. Nevertheless, ethical considerations include ensuring informed consent, protecting patient privacy and data security and facilitating equitable recruitment. Potential concerns primarily relate to ensuring consistent data quality and adherence to guidelines across different care settings. Variations in technical infrastructure and expertise between university centres and regional hospitals may initially affect the uniform implementation of treatment recommendations. As a feasibility and pilot study, MISSION4Sax does not include a control group and is not designed to provide confirmatory evidence of clinical effectiveness. Accordingly, analyses are primarily descriptive and exploratory, and observed changes cannot be causally attributed to the intervention. The small sample size, heterogeneity of tumour entities, and simultaneous implementation of all intervention components (patient pathways, CoSITuB, FDNs, training, and registry) further preclude conclusions regarding the effectiveness of individual elements.

The inclusion criteria, recruitment strategy, and outcome measures were developed in collaboration with external clinical partners to ensure feasibility within routine care. Prior to study initiation, partners expressed concerns regarding the additional workload associated with study participation and the potential for patients to be referred away from local providers. These considerations informed the pragmatic design but may limit the representativeness of the study population.

The retrospective analysis of patients from a defined timeframe at one pilot partner provides an opportunity to explore potential changes in the consistency of referral practices following the introduction of standardised pathway criteria. However, the retrospective nature of the historical data, potential incompleteness of records, and restriction to a single site limit comparability with the prospective MISSION4Sax cohort and preclude generalisability to other pilot sites or the wider Eastern Saxony region.

These limitations are inherent to the pilot nature of the project but will provide valuable insights for planning a future effectiveness study. Such a study should incorporate more strictly operationalised eligibility criteria and outcome measures, prospective screening procedures, an appropriate comparison group, and, where feasible, phased implementation or controlled designs to better evaluate the contribution of individual intervention components.

The project holds potential for long-term improvements in oncological care in medically underserved regions. By standardising care processes and integrating regional data into a database, it aims to shorten treatment delays and enhance decision-making. The collaborative framework could serve as a model for strengthening inter-institutional cooperation, particularly in the context of the ongoing hospital reform in Germany (Spreider et al. 2024). If successful, the project could serve as a basis for future regional cancer care models and support broader integration of innovative surgical and clinical research options into routine practice.

Future implementation and potential expansion of the network may be challenged by the heterogeneity of hospital information systems across participating institutions, underlining the importance of standardised datasets and interoperable electronic patient records (ePA) for the coordination of complex oncological care. Beyond their role in data acquisition, FDNs may facilitate communication and coordination between participating centres, thereby supporting implementation of the network. In addition, incorporating the patient perspective further highlights the potential impact of the project, not only through shorter travel distances and reduced waiting times, but also by strengthening trust in local healthcare providers while maintaining access to the expertise and innovative treatment options of the Comprehensive Cancer Center. Nevertheless, future evaluations should carefully assess the long-term sustainability of the network as well as differences in recruitment capacity across participating centres.

## Data Availability

The datasets generated during the study will be available from the corresponding author upon reasonable request.
